# Intent to Participate in Future Cervical Cancer Screenings Is Lower when Satisfaction with the Decision to Be Vaccinated Is Neutral

**DOI:** 10.1371/journal.pone.0098665

**Published:** 2014-06-10

**Authors:** Natalie Marya Alexander, Diane Medved Harper, Johanna Claire Comes, Melissa Smith Smith, Melinda Ann Heutinck, Sandra Martin Handley, Debra Ann Ahern

**Affiliations:** 1 Department of Community and Family Medicine, University of Missouri Kansas City School of Medicine, Kansas City, Missouri, United States of America; 2 Department of Obstetrics and Gynecology, University of Missouri Kansas City School of Medicine, Kansas City, Missouri, United States of America; 3 Department of Biomedical and Health Informatics, University of Missouri Kansas City School of Medicine, Kansas City, Missouri, United States of America; 4 Department of Family Medicine, Kansas City University of Medicine and Biosciences, Kansas City, Missouri, United States of America; 5 University of Missouri-Kansas City Student Health and Wellness, University of Missouri-Kansas City, Kansas City, Missouri, United States of America; State University of Maringá/Universidade Estadual de Maringá, Brazil

## Abstract

**Background:**

HPV vaccination programs have adversely affected participation in future cervical cancer screening. The purpose of this study is to determine the influence of decision satisfaction with accepting/rejecting the HPV vaccine, as well as traditional clinical factors, on the intent to participate in future screening.

**Methods and Findings:**

From January 2011 through August 2012 women 18–26 years old presenting for health care in an urban college student health and wellness clinic in the US Midwest were asked to complete a descriptive and medical history survey including a six element decisional satisfaction survey scored on 5-point Likert scales, where the intent to participate in future cervical cancer screening was measured. Of the 568 women who completed the decisional satisfaction survey, 17% of those <21 years and 7% ≥21 years indicated no intent to participate in future cervical cancer screenings. Among women of current screening age, the univariate risk factors of race/ethnicity, contraceptive use, number of lifetime sexual partners, and receipt of HPV vaccine were not predictors of intent for future cervical cancer screening. Instead, only a history of a prior Pap test was a significant positive predictor and only a decisional satisfaction of ‘neutral’ (Likert score = 3) for any of the four decisional satisfaction elements was a significant negative predictor. For the decisional satisfaction element “best for me personally”, there was a 78% decreased likelihood of intending to participate in future screening if the satisfaction was neutral rather than firm (aOR = 0.22, 95% CI: 0.05–0.91) and a 26 fold increased likelihood if she had had a prior Pap test (aOR = 26, 95% CI: 5–133).

**Conclusions:**

HPV vaccination implementation programs must help women be the owner of their decision around HPV vaccination and understand the importance of future participation in cervical cancer screening.

## Introduction

Cervical cancer screening guidelines continue to evolve as the role of human papillomavirus (HPV) testing develops. The US Preventive Services Task Force (USPSTF), the American College of Obstetricians and Gynecologists (ACOG), and a consensus group including the American Cancer Society (ACS) continue to emphasize that both average risk and high risk women benefit from regular screening [1]–[4]. The Australian age threshold to initiate screening is 18 years or two years after the initiation of sexual intercourse. The US threshold is older at 21 years irrespective of age of sexual debut, still lower than most European guidelines where screening starts at 25 or 30 years [5], [6]. Past experience in the Finnish population indicates that when 80% of the 25–30 year old women from 1985–2000 stopped attending screening, the incidence of cervical cancer increased five-fold within 10 years [7], [8].

The advent of HPV vaccines does not alter the recommendations for cervical cancer screening as no prophylactic vaccine is able to prevent all anogenital HPV infections, nor is there sufficient evidence that duration of efficacy will be long enough to prevent actual cancers [5], [7]. Therefore, if sufficient numbers of vaccinated women fail to participate in future cervical cancer screening, despite HPV vaccination, cervical cancer incidence will increase [9], [10]. The UK HPV immunization program reports the first study of cervical cancer screening follow ups in women now 20–22 years old wherein only 8% of women who partially completed all three doses of vaccine and only 18% of women completing all three doses presented for cervical cancer screening [11]. The Australian HPV vaccination program reports a similar lack of screening participation among both the vaccinated and unvaccinated 20–24 year olds with the smallest participation rate among those vaccinated [12].

Most vaccination programs are instituted at a parental level omitting the person actually being vaccinated from the decision process. Adolescent and young adult vaccinations provide many participants the opportunity to join in the decision making process. The Holmes Rovner Decisional Satisfaction survey applied to the decision of HPV vaccination among women 18–26 years showed that significantly more young women who have not chosen to be vaccinated are neutral vs satisfied or dissatisfied with the decision that the HPV vaccine is best for her personally, was consistent with her personal values, was hers to make and overall was a satisfactory decision [13]. While the effect of HPV vaccines on cancer prevention will not be known for decades, cervical cancer prevention still relies on participation in the organized screening program regardless of HPV vaccination. Decisional neutrality about HPV vaccination may also be associated with lack of future participation in cervical cancer screening.

The aim of this paper is to determine the influence of decisional satisfaction around HPV vaccination on the intent to participate in future cervical cancer screening among college aged US women who have and have not chosen to be vaccinated.

## Methods

This prospective study was approved by the University of Missouri Kansas City (UMKC) Social Sciences Adult Institutional Review Board (SSIRB) (#SS10-56X) as an exempt study not requiring verbal or written consent and is part of a 601 person study on how women decide to accept or reject the HPV vaccination [13].

Women aged 18–26 years old seeking care at an urban university student health and wellness clinic were invited to participate in this survey over five academic semesters from January 2011 through August 2012. The income levels of the students varied widely from those with no outside employment or source of income to those with outside employment or still living at home under their parents’ income.

The office receptionists, medical staff and providers were given scripts for study recruitment. Women could only participate once and provided self-reported descriptors and medical history including the lifetime number of sexual partners, age at first intercourse, race/ethnicity, gravidity, income, oral contraceptive use (current vs. never), barrier contraception condom use, history of prior Pap testing, abnormal Pap result, colposcopy, HPV infection, genital warts or other sexually transmitted infections. In addition, women self-reported past receipt of none, one or more doses of any HPV vaccine.

Cervical cancer prevention education was provided in a decision aid paper-based format [13], [14] where the benefits and harms of screening and HPV vaccination as published in the literature were presented [15]. A decisional satisfaction survey about the decision to accept or reject HPV vaccination was designed based on the Holmes-Rovner model [16] and included six elements assessed on a 5-point Likert scale (1 = strongly disagree, 5 = strongly agree). The decisional satisfaction survey has been validated by Holmes Rovner and used for many medical decisions. The six elements are “I am informed about the issues around cervical cancer prevention and screening”; “I expect to be vaccinated before I leave the office, if I have not already been”; “I am satisfied that my decision was consistent with my personal values”; “I am satisfied that this was my decision to make”, I am satisfied with my decision”; and “the decision I made was the best decision possible for me personally”. High scores indicated high satisfaction with the decision. Decisional neutrality is a score of 3, neither agreeing nor disagreeing with the decision to be vaccinated, potentially indicating an ambivalence or uncertainty about whether to accept or reject the HPV vaccine. Decisional neutrality is equivalent to a “I don’t know” answer. The intent to participate in future cervical cancer screening was assessed as a dichotomous choice (yes/no). If the woman was unable to decide between yes/no, she could leave the question blank.

Our past work has shown that decisional neutrality about receiving HPV vaccination identifies a young adult target population seeking more information prior to vaccination to move their decisional satisfaction away from neutral [13]. While physician recommendation has been studied as an important factor to increase HPV vaccination rates among young adolescents [17], once adult decision making abilities have developed, simple paternalistic physician recommendation is insufficient [18]. Cervical cancer screening recommendations have been a mainstay of preventive medicine, but have become muddled with the frequent changes in age of initiation, intervals and testing modalities [19]. Building on our prior work [13], we included decisional neutrality about HPV vaccination as a potential influence on future intent to participate in cervical cancer screening among college women 18–26 years old.

The six decisional satisfaction elements were explored and a subset of significant elements was used to create a decisional satisfaction summary score. Four of the six decisional satisfaction elements showed differences in decisional neutrality between the intent vs. no intent to participate in future cervical cancer screening groups. The four elements were: “The decision I made was the best decision possible for me personally”, “I am satisfied that my decision was consistent with my personal values”, “I am satisfied that this was my decision to make” and “I am satisfied with my decision”. The two elements not included in the decisional satisfaction summary score were those that were not significantly different between intent to participate in future screening groups: “I feel informed about all issues of cervical cancer screening” and “I intend to be vaccinated at this visit”.

Each of the four decisional satisfaction elements was coded in a dichotomous manner as neutral or not neutral. The score was 0 if neutral (decisional satisfaction = 3, neutral) or 1 if firm (strongly agree (5)/agree (4)/disagree (2)/strongly disagree (1)). It is important to include both the “agree” and “disagree” extremes in the “firm” decisional satisfaction category because while reflecting diametric opposites, the women are less likely to be able to move away from any anchor position to contemplate a behavior change [20].

The decisional satisfaction summary score was created from summing the four dichotomous satisfaction elements and ranged from 0–4. A score of 0 means that she was neutral in all four decisional elements around HPV vaccination; a score of 4 indicates that she was firm in all aspects of her decision around HPV vaccination whether that be in favor of or against vaccination for each decisional element.

## Statistics

Descriptive statistics included means testing with Student’s t-test and one way analysis of variance (ANOVA) followed by a two-sided post hoc Tukey test for significance testing. Chi-square comparison and Fisher’s exact testing was used for proportion comparisons as appropriate. Statistical significance was reached with a p-value <0.05. Binary logistic regression was used to predict the intent to participate in cervical cancer screening and was reported with 95% confidence intervals. Statistica version 12.0 was used for all analyses [21].

Multivariate modeling that included descriptive risk factors pertinent to intentions to participate in future cervical cancer screening were included with each of the four decisional elements about satisfaction with the HPV vaccine decision, and finally with the summary decisional score.

## Results

The decisional satisfaction survey was completed by 95% of the study subjects (568/601). Overall, 11% (60/568) of women surveyed had no intent to participate in future cervical cancer screening and 2.7% (16/601) did not answer the question. The intent to not participate in future cervical cancer screenings was divided 17% (36/212) among women <21 years and 7% (24/356) for women ≥21 years. Table 1 shows the comparison between those who intended or not to participate in future cervical cancer screening by risk factor.

**Table 1:** Subject descriptors by age group and intent to participate in cervical cancer screening.

	<21 years					≥21 years				
	N = 212					N = 356				
	Intent to Participate inFuture Cervical CancerScreening		No Intent to Participatein FutureCervical CancerScreening			Intent to Participatein Future CervicalCancer Screening		No Intent to Participatein Future CervicalCancer Screening		
	N = 176		N = 36			N = 332		N = 24		
	(83%)		(17%)			(93%)		(7%)		
	N	Mean (SD)	N	Mean (SD)	p-value	N	Mean (SD)	N	Mean (SD)	p-value
Age at first intercourse,years	143	16.5 (1.7)	16	16.6 (1.5)	NS	291	17.4 (2.1)	11	18.2 (2.8)	NS
Lifetime numberof sexual partners	164	3.3 (4.9)	29	1.8 (2.3)	NS	305	4.8 (5.7)	19	2.1 (2.9)	**0.042**
	**N**	**%**	**N**	**%**		**N**	**%**	**N**	**%**	
Race/Ethnicity										
White	105	59.7	16	44.4	NS	233	70.2	11	45.8	**0.013**
Black	44	25.0	7	19.4	NS	49	14.8	6	25.0	NS
Hispanic	5	2.8	2	5.6	NS	14	4.2	0	0.0	NS
Asian	12	6.8	7	19.4	**0.016**	23	6.9	6	25.0	**0.002**
Other	10	5.7	4	11.1	NS	13	3.9	1	4.2	NS
Gravidity										
n = 0	162	97.6	30	96.8	NS	272	85.5	20	100.0	**0.049**
n≥1	4	2.4	1	3.2		46	14.5	0	0.0	
Income										
<$10K	54	32.5	8	25.0	NS	126	38.4	18	78.3	**<0.001**
≥$10K	112	67.5	24	75.0		202	61.6	5	21.7	
Oral Contraceptive use										
No	96	54.9	27	77.1	**0.015**	146	45.5	19	79.2	**0.001**
Yes	79	45.1	8	22.9		175	54.5	5	20.8	
Condom use										
No	48	28.4	21	65.6	**<0.001**	118	37.2	13	61.9	**0.028**
Yes	120	71.0	10	31.3		195	61.5	8	38.1	
Sometimes†	1	0.6	1	3.1		4	1.3	0	0.0	
Received at least onedose of HPV vaccine										
No	73	41.7	21	58.3	NS	168	50.8	20	83.3	**0.002**
Yes	102	58.3	15	41.7		163	49.2	4	16.7	
History of priorPap test										
No	89	50.9	32	91.4	**<0.001**	31	9.6	19	82.6	**<0.001**
Yes	86	49.1	3	8.6		292	90.4	4	17.4	
History of priorabnormal Pap test										
No	158	90.3	34	97.1	NS	240	74.1	22	91.7	NS
Yes	17	9.7	1	2.9		84	25.9	2	8.3	
History of priorcolposcopy										
No	171	97.7	35	100.0	NS	273	84.3	24	100.0	**0.035**
Yes	4	2.3	0	0.0		51	15.7	0	0.0	
History of priorHPV infection										
No	169	96.6	35	100.0	NS	283	87.3	24	100.0	**0.044**
Yes	6	3.4	0	0.0		41	12.7	0	0.0	
History of genital warts										
No	173	98.9	35	100.0	NS	311	96.0	24	100.0	NS
Yes	2	1.1	0	0.0		13	4.0	0	0.0	
History of other STIs										
No	155	88.6	34	97.1	NS	288	88.9	23	95.8	NS
Yes	20	11.4	1	2.9		36	11.1	1	4.2	

Percentages are within intent to Pap grouping.

† excluded from analyses.

Bold indicates statistical significance.

Of those intending to participate in future cervical cancer screening among the <21 years and ≥21 year old age groups, 51% and 10% had not yet initiated any cervical cancer screening, respectively. 9% and 17%, respectively, had participated in cervical cancer screening in the past but indicated no intention to participate in future screening.

Of those intending to participate in future cervical cancer screening, 58% and 49% had received at least one dose of HPV vaccine among the <21 years and ≥21 year old age groups, respectively. 58% and 83%, respectively, had not yet received any HPV vaccine and did not intend to participate in future screenings.

Mean age of first intercourse was not significantly different by future intent of cervical cancer screening. Lifetime number of sexual partners was only different among women ≥21 years: those women who intend to participate in future screening had significantly more partners than those who did not intend to participate in future screening (mean 4.8 (SD 5.7) vs. 2.1 (2.9), p = 0.042).

In the ≥21 year old age group, a greater proportion of white women intended to participate in cervical cancer screening than not participate (70% vs 46%, p = 0.013). Similarly, a greater proportion of the ≥21 year old women who were multigravid (15% vs. 0%, p = 0.049); had incomes more than $10,000/year (62% vs. 23%, p<0.001); had received at least one dose of HPV vaccine (49% vs. 17%, p = 0.002); had a history of prior colposcopy (16% vs 0%, p = 0.035) and history of prior HPV infection (13% vs 0%, p = 0.044) were intending to participate in future cervical cancer screening vs. not participate.

In both age groups, a greater proportion of Asian women did not intend to participate in future cervical cancer screening than participate (19% vs 7%, p = 0.0161 and 25% vs. 75%, p = 0.002, younger and older age groups, respectively). Conversely, those who were intending to participate in future screening vs. not participate from both age groups were those who used oral contraceptives (45% vs. 23%, p = 0.015 and 55% vs. 21%, p = 0.001, respectively); used condoms (71% vs. 31%, p<0.001 and 62% vs. 38%, p = 0.028, respectively); and had a history of prior Pap testing (49% vs 9%, p<0.001 and 90% vs 17%, p<0.001, respectively).

In both age groups univariate regression modeling (Table 2) showed that the only significant predictors of intent to participate in future screenings were women who used oral contraceptives or condoms or had a prior Pap test. Asian women in both age groups, on the other hand, were less likely to intend to participate in future cervical cancer screening than white women (younger: OR = 0.26, 95% CI: 0.09–0.76 and older: OR = 0.18, 95% CI: 0.06–0.53).

**Table 2:** Univariate predictors of positive intent to participate in cervical cancer screening by age group by demographics.

	Women <21 years			Women ≥21 years		
	OR	95% CI		OR	95% CI	
Age, years	1.28	0.82	2.00	1.20	0.91	1.59
Age at first intercourse,years	0.95	0.70	1.30	0.83	0.64	1.09
Lifetime number ofsexual partners	1.19	0.98	1.45	**1.31**	**1.04**	**1.65**
Gravidity						
n = 0	1.00					
n≥1	0.74	0.08	6.86	*		
Race/ethnicity						
White	1.00			1.00		
Black	0.96	0.37	2.49	0.39	0.14	1.09
Asian	**0.26**	**0.09**	**0.76**	**0.18**	**0.06**	**0.53**
Income						
<$10K	1.00			1.00		
≥$10K	0.69	0.29	1.64	**5.77**	**2.09**	**15.93**
Oral Contraceptive Use						
No	1.00			1.00		
Yes	**2.78**	**1.20**	**6.45**	**4.55**	**1.66**	**12.50**
Condom Use						
No	1.00			1.00		
Yes	**5.25**	**2.30**	**11.97**	**2.69**	**1.08**	**6.67**
Received at least onedose of HPV vaccine						
No	1.00			1.00		
Yes	1.96	0.95	4.05	**4.85**	**1.62**	**14.50**
History of priorPap Test						
No	1.00			1.00		
Yes	**10.31**	**3.04**	**34.91**	**44.74**	**14.31**	**139.90**
History of priorabnormal Pap test						
No	1.00			1.00		
Yes	3.66	0.47	28.43	3.85	0.89	16.72
History of Other STIs						
No	1.00			1.00		
Yes	4.39	0.57	33.82	2.87	0.38	21.93

*all women with at least one pregnancy intended to continue cervical cancer screening.

Bold font indicates statistical significance.

Among the younger women, receiving the HPV vaccine was not a significant predictor for intent to participate in future cervical cancer screening.

Women ≥21 years with higher numbers of lifetime sexual partners, income greater than $10,000 per year and having received at least one dose of HPV vaccine were significantly more likely to intend to participate in future cervical cancer screening.

Decisional satisfaction for the six elements is displayed in **Table 3** by age and intent to participate in future cervical cancer screening. The decisional satisfaction element “I expect to be vaccinated before I leave the office” among those who had not already been vaccinated had 32% and 36% “disagreement” rankings for the two age groups, under 21 years and 21 and older years, respectively. All other decisional elements had few “disagreement” responses. High decisional satisfaction with “knowledge about cervical cancer prevention and screening” occurred in 66–86% of women, with no significant differences between intent to participate in future screening groups.

**Table 3:** Decisional satisfaction by age group and intent to participate in cervical cancer screening.

	< 21 years						≥21 years						
	N = 212						N = 356						
	Intent to Continue Pap Testing		No Intent to Continue Pap Testing				Intent to Continue Pap Testing			No Intent to Continue Pap Testing			
	N = 176		N = 36				N = 332			N = 24			
	N	%	N	%	p-value		N	%		N	%		p-value
I am informed about the issues aroundcervical cancer prevention and screening.													
Strongly Disagree/Disagree	10	5.7	3	8.3	NS	24		7.2		3	12.5		NS
Neutral	19	10.8	5	13.9	NS	24		7.2		4	16.7		NS
Strongly Agree/Agree	147	83.5	28	77.8	NS	284		85.6		17	65.8		NS
I expect to be vaccinated before I leave theoffice, if I have not already had one of the vaccines.													
Strongly Disagree/Disagree	49	68.0	18	85.7	NS	114		67.8		13	68.4		NS
Neutral	18	25.0	3	14.3	NS	38		22.6		4	21.1		NS
Strongly Agree/Agree	5	7.0	0	0	NS	16		9.6		2	10.5		NS
**I am satisfied that my decision was consistent with my personal values.**													
Strongly Disagree/Disagree	7	4.0	0	0	NS	9		2.7		1	4.4		NS
Neutral	**14**	**8.1**	**8**	**22.2**	**0.012**	**32**		**9.7**		**6**	**26.1**		**0.014**
Strongly Agree/Agree	152	87.9	28	77.8	NS	**289**		**87.6**		**16**	**69.5**		**0.015**
**I am satisfied that this was my decision to make.**													
Strongly Disagree/Disagree	6	3.5	3	8.5	NS	9		2.7		1	4.4		NS
Neutral	15	8.7	4	11.5	NS	**18**		**5.5**		**7**	**30.4**		**<0.001**
Strongly Agree/Agree	151	87.8	28	80.0	NS	**300**		**91.8**		**15**	**65.2**		**<0.001**
**I am satisfied with my decision.**													
Strongly Disagree/Disagree	6	3.5	1	2.9	NS	13		3.9		1	4.4		NS
Neutral	**14**	**8.1**	**7**	**20.0**	**0.034**	**32**		**9.7**		**6**	**26.1**		**0.014**
Strongly Agree/Agree	152	88.3	27	77.1	NS	**285**		**86.4**		**16**	**69.5**		**0.028**
**The decision I made was the best decision possible for me personally.**													
Strongly Disagree/Disagree	7	4.0	0	0	NS	**14**			**4.2**	**3**		**13.6**	**0.045**
Neutral	**23**	**13.1**	**11**	**30.6**	**0.010**	**43**			**13.0**	**7**		**31.8**	**0.014**
Strongly Agree/Agree	145	82.9	25	69.4	NS	**275**			**82.8**	**12**		**54.6**	**0.001**

Bold decisional elements are those that showed a statistically significant difference in the proportion of neutral responses between those intending to be screened and those with no future intentions of participating in cervical cancer screening.

The significant four decisional satisfaction elements had greater proportions of neutral scores among those ***not*** intending to participate in future cervical cancer screening than intending to participate for at least one of the age groups. For example, among women <21 years, for the decisional element “the decision about whether to have the HPV vaccination is consistent with my personal values”, 22% responded neutrally and did ***not*** intend to participate in future cervical cancer screening vs. 8% who were neutral responders and intended to participate in future cervical cancer screening (p = 0.012).

For the same decisional element (“consistent with my personal values”), among women ≥21 years, 26% responded neutrally and did ***not*** intend to participate in future cervical cancer screenings vs. 10% who were neutral responders and intended to participate in future cervical cancer screenings (p = 0.014).

Table 3 shows that the decisional satisfaction with the four significant elements was anchored at agreement rather than disagreement. Nonetheless, a firm responder was defined using both anchors of agreement and disagreement where rankings were classified as either a 1/2/4/5 choice on the Likert scale. A neutral responder continued to be defined as a Likert score of 3. Table 4 presents the univariate predictors of intent to participate in cervical cancer screening by significant decisional satisfaction elements where neutrality was compared to a firm response (agreeing or disagreeing).

**Table 4:** Predictors of intent to participate in cervical cancer screening.

	Women <21 years		Women ≥21 years	
Decisional Neutrality Elements	OR	95% CI	OR	95% CI
This decision is best for me personally				
Firm*	1.00		1.00	
Neutral	**0.34**	**0.15, 0.79**	**0.34**	**0.13, 0.87**
This decision is consistent with my personal values				
Firm*	1.00		1.00	
Neutral	**0.31**	**0.12, 0.80**	**0.30**	**0.11, 0.83**
I am satisfied that the decision was mine to make				
Firm*	1.00		1.00	
Neutral	0.74	0.23, 2.38	**0.13**	**0.05, 0.36**
I am satisfied with my decision				
Firm*	1.00		1.00	
Neutral	**0.35**	**0.13, 0.96**	**0.30**	**0.11, 0.83**
**Decisional Satisfaction Summary Score** †				
4	1.00		1.00	
3	0.79	0.21, 2.96	0.30	0.09, 1.00
2	0.32	0.10, 1.01	0.36	0.07, 1.75
1	**0.16**	**0.04, 0.59**	0.29	0.06, 1.44
0	1.11	0.13, 9.45	**0.10**	**0.03, 0.38**

Bold numbers indicate statistical significance.

*Firm decision means the satisfaction score for the category was strongly agree/agree/disagree/strongly disagree.

† The summary score was created from the sum of four dichotomous satisfaction categories (“personally”, “my personal values”, “mine to make” and “satisfied”) where the category was a 0 if neutral or a 1 if firm (strongly agree/agree/disagree/strongly disagree). Decisional satisfaction summary scores ranged from 0–4; 0 means that all four elements were ranked as neutral and 4 means that none of the four elements were ranked as neutral.

For younger women <21 years old, three elements of satisfaction around their HPV vaccine decision predicted a significantly lower intent for future participation in cervical cancer screening if the satisfaction with the element was neutral rather than firmly anchored: the decision that HPV vaccine was “best for me personally”, the decision was “consistent with my values”, and they were “satisfied” with the decision to be vaccinated. For instance, those who were neutral to “the decision was best for me personally” expressed a significantly lower likelihood (OR = 0.34 95% CI: 0.15, 0.79) of participation in future cervical cancer screening than those who were firm. When the definition of firm decision was changed to just reflect the agreement scores, 4/5, there was no change in the significant predictors identified (data not shown).

Additionally, for these young women the decreased likelihood of future screening was predominantly in the group of younger women who had not yet received any HPV vaccine and were ***neutral*** vs. firm in their decisional satisfaction on two decisional elements around HPV vaccination: the decision is” best for me personally” and the decision is “consistent with my personal values” (OR = 0.26 95% CI: 0.09, 0.78 and OR = 0.27 95% CI: 0.08, 0.90, respectively).

For women ≥21 years old, being decisionally neutral as opposed to firm for each of the four elements of the decision around HPV vaccination was also associated with a significant decrease in the likelihood of participating in future cervical cancer screening. For instance, women 21 years or older who were decisionally neutral regarding their decision that HPV vaccination was “best for me personally” indicated that they were also significantly less likely (OR = 0.34, 95% CI: 0.13, 0.87) to participate in future cervical cancer screenings than women who were firm in their opinion that HPV vaccination was best for them personally.

In addition, women 21 years and older who had not been vaccinated with one of the HPV vaccines indicated a significantly lower likelihood (OR = 0.16, 95% CI: 0.05, 0.46) of participating in future cervical cancer screening if they were neutral vs firm in their satisfaction that the decision to be vaccinated was theirs to make. In other words, if they did not personally take ownership of the decision to be vaccinated, they indicated a lower intent to participate in future screening.

The final univariate model used the decisional satisfaction summary score as a predictor of intent to participate in future cervical cancer screenings. As the number of neutral rankings of decisional elements increases, women indicate that they have an increasingly lower likelihood of future cervical cancer screening intent (p for trend <0.05). When women <21 years are decisionally neutral in three of the four decisional elements, they have significantly lower intentions of participating in future cervical cancer screening than those with no decisionally neutral elements (OR = 0.16, 95% CI: 0.04, 0.59). Among women 21 years and older, the chances of intending to participate in future screening are 90% less likely if they were neutral in their decisional satisfaction on all four elements than if they had no neutral decision elements (OR = 0.10, 95% CI: 0.03, 0.38).

The multivariate models (Table 5) incorporate the significant clinical risk factors from the univariate models with the elements of decisional satisfaction to predict the intent to participate in future cervical cancer screening. Five models were created for each age group to evaluate the four decisional elements independently followed by the summary decisional satisfaction score; each model included clinical risk factors.

**Table 5:** Multivariate Models predicting Intent to Participate in Cervical Cancer Screening for Women ≥21 years.

	Model 1				Model 2		Model 3			Model 4		Summary Model						
	aOR	95% CI			aOR	95% CI	aOR	95% CI		aOR	95% CI	aOR					95% CI	
N of lifetime sex partners	1.01	0.81–1.27			1.00	0.80–1.28	1.01	0.80–1.27		1.02	0.83–1.24	1.19					0.94–1.50	
Race																		
White	1.00				1.00		1.00			1.00		1.00						
Black	0.38	0.08–1.92			0.34	0.07–1.82	0.34	0.06–1.87		0.32	0.06–1.56	0.56					0.14–2.25	
Asian	0.32	0.06–1.77			0.20	0.04–1.04	0.31	0.05–1.76		0.26	0.05–1.42	**0.18**					**0.04–0.79**	
Income																		
<$10,000	1.00				1.00		1.00			1.00		1.00						
≥$10,000	3.09	0.81–11.78			**4.04**	**1.02–16.10**	2.62	0.68–10.15		3.28	0.86–12.53	**6.19**					**1.74–22.00**	
Oral Contraceptive Use																		
No	1.00				1.00		1.00			1.00		1.00						
Yes	1.34		0.29–6.16		1.30	0.29–5.71	1.53	0.31–7.55		1.41	0.31–6.51	**4.70**					**1.17–18.92**	
History of Prior Pap test																		
No	1.00				1.00		1.00			1.00				1.00				
Yes	**25.74**		**4.98–133.1**		**26.34**	**5.17–134.2**	**31.94**	**5.73–178.2**		**25.83**	**5.19–128.5**			0.99			0.18–5.58	
Received at least one dose of HPV vaccine																		
No	1.00				1.00		1.00			1.00				1.00				
Yes	1.99		0.38–10.39		1.99	0.40–9.89	1.11	0.22–5.64		1.80	0.37–8.75			2.76			0.71–10.78	
**Decisional Neutrality Elements**																		
This decision is bestfor me personally																		
Firm	1.00																	
Neutral	**0.22**		**0.05–0.91**															
This decision is best forme personally																		
Firm	1.00																	
Neutral	**0.22**		**0.05–0.91**															
This decision is consistentwith my personal values																		
Firm					1.00													
Neutral					**0.14**	**0.03–0.68**												
I am satisfied that the decisionwas mine to make																		
Firm							1.00											
Neutral							**0.09**		**0.01–0.55**									
I am satisfied with my decision																		
Firm										1.00								
Neutral										**0.19**	**0.04–0.84**							
**Decisional Satisfaction Summary Score** †																		
4													1.00					
3													0.26					0.04–1.37
2													**0.08**					**0.01–0.64**
1													0.19					0.03–1.30
0													0.20					0.03–1.20

Bold numbers indicates statistical significance.

† The summary score was created from the sum of four dichotomous satisfaction categories (“personally”, “my personal values”, “mine to make” and “satisfied”) where the category was a 0 if neutral or a 1 if firm (strongly agree/agree/disagree/strongly disagree). Decisional satisfaction summary scores ranged from 0–4. A score of 0 means that all four elements of decisional satisfaction were neutral, a score of 1 means that three elements of decisional satisfaction were neutral, a score of 2 means that 2 elements of decisional satisfaction were neutral, a score of 3 means that one element of decisional satisfaction was neutral, and a score of 4 means that no elements of decisional satisfaction were neutral.

In women too young for current screening (those under 21 years of age), only Asian race compared to White race was a significant predictor of 80–85% reduced likelihood of intent to participate in future screening (data not shown).

In women of screening age, whose intent to participate in future screenings has immediate consequences for early cancer detection and treatment, the first four models included both a single decisional satisfaction element and significant clinical risk factors (Table 5). Race, contraceptive use, number of lifetime sexual partners and receipt of HPV vaccine were not predictors of intent for future cervical cancer screening. Instead, history of a prior Pap test was a positive predictor, and being neutral in each of the decisional elements was a negative predictor of intent for future screening. For only the decisional satisfaction element “consistent with my personal values”, low income level became significant in addition to the history of prior Pap testing as large positive predictors.

The fifth multivariate model was the summary model, using the summary decisional satisfaction score and significant clinical risk factors from univariate analyses. This model resulted in two positive and two negative predictors for intent to participate in future cervical cancer screening: the positive predictors were contraceptive use and higher income; the negative predictors were Asian race and being neutral in any two of the four decisional elements.

## Discussion

Our work shows that in a young adult population, despite attending college (i.e. being well-educated), those who have multiple neutral decisional satisfaction scores around HPV vaccination are at the highest risk for forgoing future cervical cancer screenings. The influence of decisional neutrality around the elements of the HPV vaccination decision retained significance even after considering other reproductive health factors, such as gravidity, need for contraception, and number of lifetime sexual partners. The ambivalence to HPV vaccination and the decreased intended likelihood of future screening makes this group of women at high risk for cervical cancer screening abandonment regardless of age.

Ideally, vaccinated women will participate in future cervical cancer screenings so that there is little chance of increasing the incidence of cervical cancer [5], [7], [10]. The worst public health outcome would be for women to choose not to be vaccinated and not to present for screening [22]–[25]. Actual follow up data on women 20–22 years of age sampled after the 2008 UK HPV National Immunization program shows that only 49% of women, regardless of presentation for cervical cancer screening, received at least one dose of HPV vaccine; only 46% of women, regardless of vaccine receipt, presented for cervical cancer screening; and only 26% of women with at least one dose of HPV vaccine presented for cervical cancer screening [11]. These data are also reflected in Australian women 20–24 years old where only 40% of HPV vaccinated and 46% of unvaccinated women presented for cervical cancer screening [12]. These significant lapses in cervical cancer screening are worrisome regardless of HPV vaccine receipt and, while our study measured intent for future participation and not actual participation, these lapses in screening are indicated to happen in the future in our study as well.

Healthy People 2020 aims for a 93% cervical cancer screening participation rate, up from a current self-reported (National Health Interview Survey (NHIS)) baseline rate of 85% [26]. Actual cervical cancer screening rates show an average of 76% of women with commercial insurance, and 70% of women in Medicaid plans [27]. Our work shows that among women 21 years and older enrolled in an urban college, 93% intend to participate in future screening, dropping to 83% among those younger than 21 years old. Our population’s intent to screen rate appears to meet current US national rates and goals.

Our multivariate models showed that actual HPV vaccine receipt was not a significant positive or negative predictor of women’s intentions for future cervical cancer screening, providing reassurance that cervical cancer screening rates should not decrease. On the other hand, while actual HPV vaccination may not lead to a decrease in intent for future screening among the vaccinated, neither did our data indicate that those not choosing HPV vaccination would participate in future cervical cancer screening. Specifically, our data show that there is a small, but worrisome subgroup of college educated women who have no intention of participating in future cervical cancer screening and have chosen not to be vaccinated against HPV. This subgroup also does not use contraceptives or condoms, thus avoiding those health care visits where cervical cancer screening is likely to be discussed. Of similar concern is the high rate of college educated US Asian women choosing both no screening and no vaccination in this study and another [28]. To compound this concern is the continued association of low income women with no intention of seeking future cervical cancer screening, regardless of HPV vaccine status, as has been shown in several studies [11], [12], [29].

Finally, we postulate that future cervical cancer prevention programs consider including an assessment of the decisional satisfaction about the HPV vaccine. The most important women to target for future screening are those who are decisionally neutral about accepting or rejecting the HPV vaccine. Our work shows that higher decisional satisfaction with the decision to accept or reject HPV vaccination is the second most prominent positive or negative driver of the intent to participate in future cervical cancer screening. The decisional neutrality is as potent a negative predictor for lack of intent to participate in future cervical cancer screening as prior Pap testing is a positive predictor. In particular, Asian women older than 21 years who express decisional neutrality for two decisional satisfaction elements are at great risk for lack of future cervical cancer screening participation.

The US advertising campaign around HPV vaccination needs to change to emphasize the importance of participation in future cervical cancer screening; and, that women who receive HPV vaccines may still have abnormal results that have to be followed up. The UK program showed that even with at least one dose of HPV vaccine, 14–15% of women who attended cervical cancer screening still had an abnormal screen [11]. While HPV vaccine studies fully acknowledge that there is only an expected 20% decrease in overall abnormal cytology [30], [31], the effect on compliance with future cervical cancer screening may be further influenced by negative social media posts about “vaccine failures” when screenings are abnormal.

In addition, being satisfied that the decision around HPV vaccination is the woman’s own decision, not passively made for or imposed on her, is a good indicator for intention to participate in future cervical cancer screening. This means that an open shared-decision making approach with full disclosure about the HPV vaccination is most important for effecting behavioral compliance with future cervical cancer screening [32].

## Limitations

This work was based in an urban college health and wellness clinic where two thirds were undergraduate students and one third self-identified as graduate or professional studies students. This highly educated study population may not reflect the actions of a more general population as is represented by the Healthcare Effectiveness Data and Information Set (HEDIS) which summarize cervical cancer screening rates, among other data, reported by all health care plans for physician performance evaluations in all populations. Similarly our data reflect decisions of young adult women, not of parents nor of adolescents. While this limitation is real, the decision to participate in future cervical cancer screening is an individual adult decision.

In addition, this population is younger than 27 years old with only 13% of those of screening age having had at least one pregnancy. Choices for screening at this age may have little resemblance to choices made when older, pregnant, or after a health adversity, but nonetheless are important at initiating preventive screening practices. The lack of intent to participate in future cervical cancer screening among women younger than 27 years may or may not result in undetected cancers depending on whether or not the lack of screening persists into the 30–39 year old age range where cervical cancer screening is most sensitive [6], [33].

Cervical cancer screening is changing to primary HPV testing with follow up or co-testing with cytology, with some centers offering a self-sampling option [3], [34]–[37]. Our study defined cervical cancer screening in the familiar sense of Pap testing, as others have found that women will indicate a decreased intent to screen if the screening test is primary HPV testing at lengthened intervals [38]. Future work will have to query whether HPV vaccination status impacts cervical cancer screening behavior when screening is not the familiar Pap testing.

## Conclusions

The intent to participate in cervical cancer screening after HPV vaccination is reassuring among those already of screening age, but decisional neutrality about HPV vaccination as opposed to firm HPV vaccine acceptance or rejection has a tremendous potential to inhibit future cervical cancer screening.
