# Correction: Intent to Participate in Future Cervical Cancer Screenings Is Lower when Satisfaction with the Decision to Be Vaccinated Is Neutral

**DOI:** 10.1371/journal.pone.0107790

**Published:** 2014-09-08

**Authors:** The PLOS ONE Staff

There are errors in Table 5. Please see the corrected Table 5 here.

**Table 5:** Multivariate Models predicting Intent to Participate in Cervical Cancer Screening for Women ≥ 21 years.

Model 1			Model 2		Model 3		Model 4		Summary Model	
aOR		95% CI	aOR	95% CI	aOR	95% CI	aOR	95% CI	aOR	95% CI
N of lifetime sex partners	1.01	0.81-1.27	1.00	0.80-1.28	1.01	0.80-1.27	1.02	0.83-1.24	1.19	0.94-1.50
Race										
White	1.00		1.00		1.00		1.00		1.00	
Black	0.38	0.08-1.92	0.34	0.07-1.82	0.34	0.06-1.87	0.32	0.06-1.56	0.56	0.14-2.25
Asian	0.32	0.06-1.77	0.20	0.04-1.04	0.31	0.05-1.76	0.26	0.05-1.42	**0.18**	**0.04-0.79**
Income										
<$10,000	1.00		1.00		1.00		1.00		1.00	
≥$10,000	3.09	0.81-11.78	**4.04**	**1.02-16.10**	2.62	0.68-10.15	3.28	0.86-12.53	**6.19**	**1.74-22.00**
Oral Contraceptive Use										
No	1.00		1.00		1.00		1.00		1.00	
Yes	1.34	0.29-6.16	1.30	0.29-5.71	1.53	0.31-7.55	1.41	0.31-6.51	**4.70**	**1.17-18.92**
History of Prior Pap test										
No	1.00		1.00		1.00		1.00		1.00	
Yes	**25.74**	**4.98-133.1**	**26.34**	**5.17-134.2**	**31.94**	**5.73-178.2**	**25.83**	**5.19-128.5**	0.99	0.18-5.58
Received at least one dose of HPV vaccine										
No	1.00		1.00		1.00		1.00		1.00	
Yes	1.99	0.38-10.39	1.99	0.40-9.89	1.11	0.22-5.64	1.80	0.37-8.75	2.76	0.71-10.78
Decisional Neutrality Elements										
This decision is best for me personally										
Firm	1.00									
Neutral	**0.22**	**0.05-0.91**								
This decision is consistent with my personal values										
Firm			1.00							
Neutral			**0.14**	**0.03-0.68**						
I am satisfied that the decision was mine to make										
Firm					1.00					
Neutral					**0.09**	**0.01-0.55**				
I am satisfied with my decision										
Firm							1.00			
Neutral							**0.19**	**0.04-0.84**		
Decisional Satisfaction Summary Score†										
4									1.00	
3									0.26	0.04-1.37
2									**0.08**	**0.01-0.64**
1									0.19	0.03-1.30
0									0.20	0.03-1.20

Bold numbers indicates statistical significance.

† The summary score was created from the sum of four dichotomous satisfaction categories (“personally”, “my personal values”, “mine to make” and “satisfied”) where the category was a 0 if neutral or a 1 if firm (strongly agree/agree/disagree/strongly disagree). Decisional satisfaction summary scores ranged from 0-4. A score of 0 means that all four elements of decisional satisfaction were neutral, a score of 1 means that three elements of decisional satisfaction were neutral, a score of 2 means that 2 elements of decisional satisfaction were neutral, a score of 3 means that one element of decisional satisfaction was neutral, and a score of 4 means that no elements of decisional satisfaction were neutral.
